# Anaphylaxis following a transvaginal ultrasound

**DOI:** 10.1186/s13223-015-0106-9

**Published:** 2016-01-22

**Authors:** Baruch D. Jakubovic, Corey Saperia, Gordon L. Sussman

**Affiliations:** Department of Medicine, Sunnybrook Health Sciences Centre, University of Toronto, 2075 Bayview Avenue-D416, Toronto, ON M4N 3M5 Canada; Division of Allergy and Immunology, Department of Medicine, St. Michael’s Hospital, Toronto, ON Canada

**Keywords:** Anaphylaxis, Polyethylene glycol, Iatrogenic reaction

## Abstract

Polyethylene glycol is a ubiquitous, water-soluble, organic compound found in a wide variety of commercially available products. While generally a benign substance, in rare instances, it can induce hypersensitivity reactions. Herein, we describe a case of anaphylaxis to polyethylene glycol-containing lubricating gel used for a transvaginal ultrasound. This case highlights the importance of early recognition of a rare cause of anaphylaxis that may occur in the health-care setting. It is of particular importance given the widespread use of similar lubricating materials in multiple practice settings for the use of internal examinations and ultrasonography.

## Background

Polyethylene glycol (PEG) is a water-soluble, organic compound included in a wide variety of food products, cosmetics, and industrial materials [1, 2]. In the healthcare setting, it is also a common ingredient in medications and procedural agents. Specific case reports have documented PEG-associated hypersensitivity or anaphylaxis following ingestion of medication tablets [1, 2] as well as following whole-bowel irrigation for colonoscopy or barium enema [3–6]. Reactions have also been described after administration of depot steroids [7, 8]. Lower doses of PEG therapy used for constipation management have been reported to cause anaphylaxis as well [9].

Anaphylactic reactions following transvaginal ultrasound procedures have also been described, but these were attributable to mucosal latex exposure from the condom-cover used for the ultrasound probe [10, 11]. Periprocedural reactions to gel-type products containing methyl celluloses and chlorhexidine have also been described [12, 13]. One brief report by Villa and colleagues from Italy, in a letter to the editor over 15 years ago attributed an anaphylactic reaction following a transvaginal ultrasound to the gel, given the patient’s negative prick test to latex [14]. The authors speculated that the most plausible explanation was gel hypersensitivity, but did not elaborate further. Herein, we describe an anaphylactic reaction post-transvaginal ultrasound attributable to the PEG contained within the lubricating gel.

## Case

Within minutes of undergoing a transvaginal ultrasound, a 41 year old female developed immediate intra and perivaginal pruritus and burning. These symptoms did not resolve with self-administered cetirizine. Over the course of several hours, the patient developed a progressive multisystem reaction including cutaneous flushing, dyspnea, cough, nasal congestion, and generalized urticaria. Severe local labial swelling and throat tightness prompted her to seek emergent care at a nearby hospital. At her initial triage assessment, vital signs were within normal limits, though diffuse urticaria without angioedema was noted. Management included intramuscular epinephrine followed by intravenous diphenhydramine, ranitidine, and corticosteroids for presumed anaphylaxis. Her symptoms subsequently abated and did not recur during or after her period of observation while at hospital. She was discharged home with a prescription for prednisone 40 mg daily for 4 days along with an epinephrine autoinjector, and was referred for further allergic evaluation.

Her past medical history was significant for cold urticaria and an ovarian cyst for which she had a prior resection and had been undergoing annual transvaginal screening examinations for the previous 18 years. She had a similar reaction 1 year prior that started with local vaginal pruritus which was later followed by periorbital and lingual swelling, urticaria, cough, and dyspnea. At that time, she had similarly presented to a local emergency department for acute management. She was a highly-active non-smoker, denied illicit drug use, and did not regularly consume alcohol. There was no personal or family history of atopy or autoimmunity. She was not taking any medications.

**Fig. 1:**
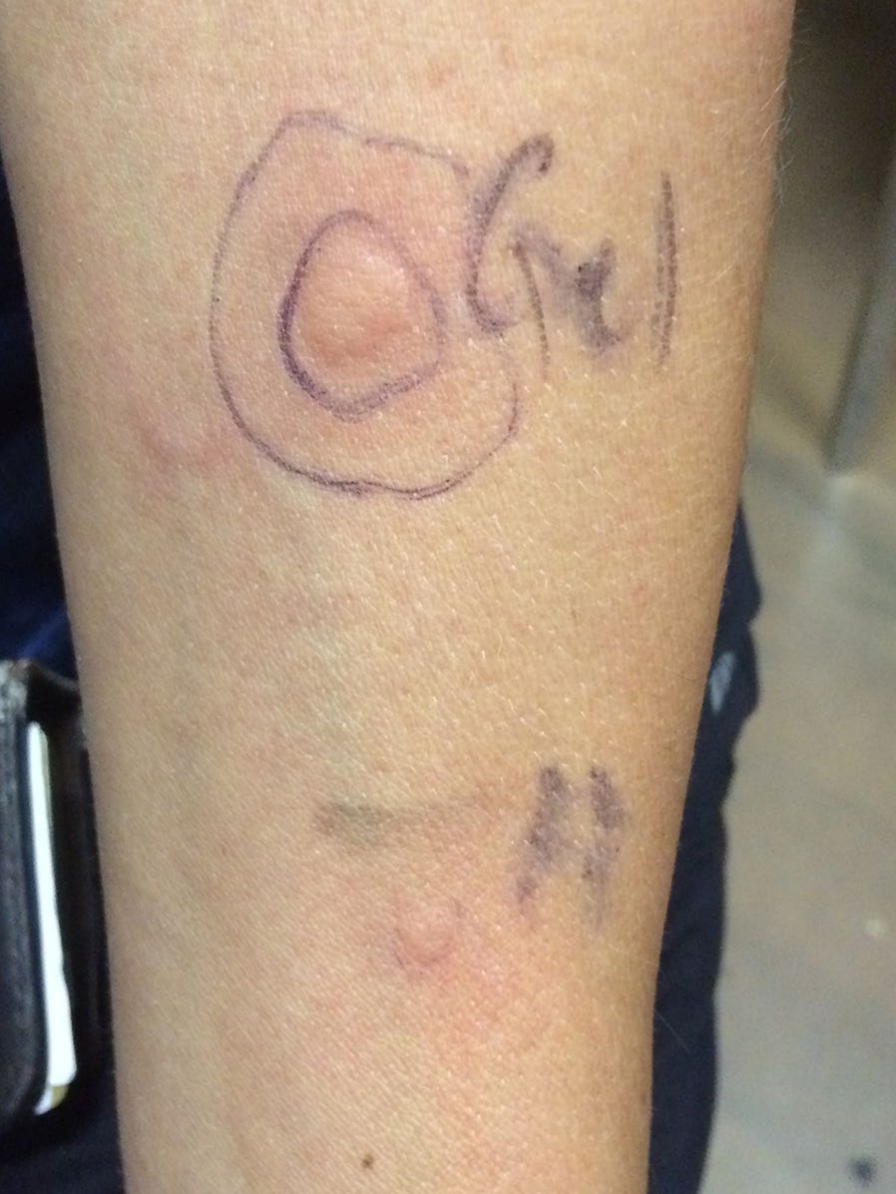
Skin prick testing result for ultrasound gel vs positive histamine control

Allergy skin testing was performed by prick method to latex (low ammoniated natural rubber latex extract), as well as to samples provided by the patient from the non-latex condom that was used to cover the ultrasound probe and to the ultrasound gel used during the procedure. Additional testing was performed to the individual gel components which were obtained from the gel manufacturer (Chester Packaging, Cincinnati, OH). These included polyethylene glycol (molecular weight 8000), glycerin, carbomer, propylparaben, methylparaben, and sodium hydroxide. At 10 min, equally strongly positive reactions measuring 12 mm were noted to two gel samples (from separate gel sachets) as well as to the polyethylene glycol sample provided by the manufacturer. These results were validated in our patient by both positive histamine and negative saline controls (Fig. 1). No specific increase in wheal size was noted in the subsequent period of time. Specific serum IgE (immunocap) testing to latex was also negative. Given a presumed IgE-mediated anaphylactic reaction to the polyethylene glycol, the patient was counselled regarding avoidance of PEG-containing substances by all routes in the future and advised to carry an epinephrine auto-injector with her at all times.

## Discussion

This case highlights two key points of relevance: firstly, severe hypersensitivity reactions, though often immediate in onset, can evolve over the course of minutes to hours. As the initial presenting symptoms may seem minor or benign in nature, clinical suspicion of an iatrogenic reaction is warranted when new symptoms of any type develop immediately following a health care exposure. Secondly, while rare, mucosal exposure to polyethylene glycol-containing products can trigger multisystem anaphylactic reactions.

Typically, anaphylactic reactions are hyperacute in nature, presenting within minutes of antigen exposure, with a rapid evolution of multisystem manifestations relating to mast-cell release. However, rarely anaphylactic reactions can develop in a more sub-acute fashion, and may develop up to 72 h following initial exposure—as can be seen with penicillin reactions [15]. Whether or not the mechanism is similar to biphasic reactions which one group suggested may be related to an extended inflammatory process triggered by the initial IgE reaction remains unclear [16]. A possible contributor in our patient may have been the presence of residual gel on the vaginal mucous membrane which led to ongoing antigenic exposure, though the actual mechanism is not known.

Gels and lubricating agents serve multiple purposes in the healthcare setting, typically in relation to procedures that involve mucous membranes. Other common gel constituents that may rarely be associated with anaphylaxis include methyl celluloses (which the gel used for this procedure also contained) and chlorhexidine [12, 13]. Periprocedural anaphylaxis in the health-care setting can also be related to the use of latex-containing gloves or barrier devices, such as condoms that are used for transvaginal ultrasounds.

As there are limited reports of PEG-associated anaphylaxis, the degree to which the administration route or molecular weight influences the nature of the reaction is not clear [17]. In addition to a suggestive history, an IgE-related mechanism is supported by positive skin prick testing to multiple PEG-containing products of varying molecular weights, positive basophil histamine release assays, and suppression of histamine release through the utilization of omalizumab and both mono- and dimeric fractions of PEGs [8, 18].

Given the presumed rarity of PEG hypersensitivity, and its presence in many different products and formulations with a myriad of exposure routes, it is unclear who might be at risk. Further complicating matters is that the development of hypersensitivity might not even be to PEG itself, but to substances that contain compounds which have moieties that resemble PEG [19]. Despite the widespread use of PEG, the prevalence of reactions attributable to PEG exposure would seem to be low. However, PEG-induced reactions may be more common than we think—and might explain cases of idiopathic anaphylaxis.

For those in whom a PEG-related allergy is suspected, one group suggested an oral challenge to identify a threshold exposure level that might enable specific guidance regarding avoidance strategies [17]. However, oral provocation in that patient resulted in a multisystem reaction that necessitated administration of antihistamine and intravenous steroid. Despite poor alimentary absorption of high molecular weight PEG solutions, low levels can be detected in the urine (implying systemic absorption) and appear to be adequate for anaphylaxis [4]. Moreover, given the rising prevalence of PEGylated drugs to help prolong drug half-life, prescribers and patients should be made aware that anaphylaxis to mucosally-exposed PEG may also increase the risk of patients reacting to PEGylated drugs, as predicted by skin prick testing [9]. Patients with anaphylaxis to PEG can thus also react to PEGylated drugs. Overall, skin prick testing is the best diagnostic modality from a safety perspective, though basophil histamine release assays can also be used. As aforementioned, despite the purported mechanism being IgE-mediated, at present there is no assay of which we are aware for the measurement of anti-PEG IgE antibodies.

As with all suspected or confirmed instances of IgE-mediated hypersensitivity, management should be strict avoidance of the culprit antigens. However, the lack of consistent labelling and identification of PEG as a chemical constituent in many products makes this a challenge [8]. Thus, obvious avoidance measures should be recommended and include laxatives and colonoscopic preparations, as well as substances used for procedures involving mucous membranes, but patients should be advised to read labels. Of note, though PEG is the most widely used agent for colonoscopic preparation, other options are available [6]. Nevertheless, avoidance strategies are laden with the potential for error, and as a result, patients with PEG allergy should carry an epinephrine autoinjector as a safety net.

## Conclusion

This case demonstrates that mucosal exposure to PEG found in lubricating gel can trigger anaphylaxis and raises the possibility that PEG may be an unrecognized allergen in the healthcare setting. All cases of anaphylaxis need a thorough investigation to identify exact causation. It cannot be assumed that all reactions are caused by more common allergens such as natural rubber latex, or alternative antigens such as methyl celluloses and chlorhexidine. Thus, healthcare sites where providers perform procedures such as internal ultrasounds or physical examinations should be aware of this rare, but potentially fatal complication of performing what are generally thought of as routine medical interventions with little risk aided by PEG-containing lubricants. Further study is needed to evaluate the prevalence of PEG hypersensitivity and to clarify who might have a heightened risk. Providers should be educated on the possibility of PEG hypersensitivity and be able to recognize the early signs of anaphylaxis in order to enable appropriate medical care, consultation, or transfer to an acute medical facility.

## Consent

Written informed consent was obtained from the patient for publication of this case report and any accompanying images.

## Abbreviation

PEG: polyethylene glycol
